# Columnar Liquid Crystals of Copper(I) Complexes with Ionic Conductivity and Solid State Emission

**DOI:** 10.3390/molecules28104196

**Published:** 2023-05-19

**Authors:** Viorel Cîrcu, Constantin P. Ganea, Mihail Secu, Doina Manaila-Maximean, George Cătălin Marinescu, Roua Gabriela Popescu, Iuliana Pasuk

**Affiliations:** 1 Department of Inorganic and Organic Chemistry, Biochemistry and Catalysis, University of Bucharest, 4-12 Regina Elisabeta Bld., Sector 5, 030018 Bucharest, Romania; 2 National Institute of Materials Physics, Atomistilor 405A, 077125 Magurele, Romania; 3 Department of Physics, University Politehnica of Bucharest, 313 Spl. Independentei, 060042 Bucharest, Romania; 4 Academy of Romanian Scientists, 3 Ilfov Str., 050094 Bucharest, Romania; 5 Asociația Independent Research, 58 Timișului, Sector 1, 012416 Bucharest, Romania; 6 Department of Biochemistry and Molecular Biology, University of Bucharest, 91-95 Spl. Independentei, 050095 Bucharest, Romania

**Keywords:** liquid crystals, copper(I), luminescence, columnar phase, ionic conductivity, dielectric spectroscopy, benzoylthiourea

## Abstract

Two neutral copper(I) halide complexes ([Cu(BTU)_2_X], *X* = Cl, Br) were prepared by the reduction of the corresponding copper(II) halides (chloride or bromide) with a benzoylthiourea (BTU, *N*-(3,4-diheptyloxybenzoyl)-*N*′-(4-heptadecafluorooctylphenyl)thiourea) ligand in ethanol. The two copper(I) complexes show a very interesting combination of 2D supramolecular structures, liquid crystalline, emission, and 1D ionic conduction properties. Their chemical structure was ascribed based on ESI–MS, elemental analysis, IR, and NMR spectroscopies (^1^H and ^13^C), while the mesomorphic behavior was analyzed through a combination of differential scanning calorimetry (DSC), polarizing optical microscopy (POM), and powder X-ray diffraction (XRD). These new copper(I) complexes have mesomorphic properties and exhibit a hexagonal columnar mesophase over a large temperature range, more than 100 K, as evidenced by DSC studies and POM observations. The thermogravimetric analysis (TG) indicated a very good thermal stability of these samples up to the isotropization temperatures and over the whole temperature range of the liquid crystalline phase existence. Both complexes displayed a solid-state emission with quantum yields up to 8% at ambient temperature. The electrical properties of the new metallomesogens were investigated by variable temperature dielectric spectroscopy over the entire temperature range of the liquid crystalline phase. It was found that the liquid crystal phases favoured anhydrous proton conduction provided by the hydrogen-bonding networks formed by the NH…X moieties (*X* = halide or oxygen) of the benzoylthiourea ligand in the copper(I) complexes. A proton conductivity of 2.97 × 10^−7^ S·cm^−1^ was achieved at 430 K for the chloro-complex and 1.37 × 10^−6^ S·cm^−1^ at 440K for the related bromo-complex.

## 1. Introduction

Over the last few years, we have seen an increase in the development and manufacture of materials for emission applications (OLEDs, luminescence-based sensors, etc.). In this respect, the search for new emitting materials have led to low-cost copper(I) complexes, which can show exceptional luminescent properties, including thermally activated delayed fluorescence (TADF) and high emission efficiencies of nearly 100%, by using both singlet and triplet excitons, despite small spin-orbit coupling [1,2,3,4,5,6,7,8]. Copper represents a cheaper alternative to that offered by platinum or iridium (commonly used in practical lighting applications) due to its higher natural abundance (27 ppm) as well as lower production costs. Most of the luminescent copper(I) complexes developed so far are based on heteroleptic tetracoordinate cationic copper(I) with phosphine ligands and diimine nitrogen-containing ligands [9,10]. Moreover, most of the reports on copper(I) complexes with sulfur donor ligands are limited to their structural aspects and their biological properties, while studies regarding their photochemical properties are scarce [11,12,13,14,15,16,17,18]. Unlike the cationic analogues, the photophysical properties of mononuclear neutral copper(I) compounds have not been well investigated. Current research indicates that the development of neutral copper(I) complexes is crucial for achieving high phosphorescent radiative rates from these materials and thus for the realization of highly-efficient emission-based devices [19,20,21]. On the other hand, luminescent liquid crystalline compounds (LC) provide anisotropic long-range order and thus polarized emission that should improve the performance parameters of electro-optical devices. Although the copper(I) ion can give complexes with low coordination numbers (2–4) suitable for the stabilization of LC phases, metallomesogens (liquid crystals based on metal complexes) composed of copper(I) complexes are quite rare, unlike other d^10^ metals. The LCs based on copper(I) complexes reported so far are limited to several classes of ligands related to alkylthiolates [22], isocyanides [23,24,25,26,27], biquinoline [28], phenantrolines, azamacrocycles, or Schiff bases derived from 2-iminopyridines [29,30], giving either mono- or dinuclear two- or tetra-coordinate complexes [31]. The cyclic trinuclear copper(I) complexes with pyrazolate type ligands show interesting emission properties and highest absolute quantum yields (42%) [32,33]. Several other structural types, including the ionic columnar metallomesogens formed by three-coordinate copper(I) complexes with bis(1-pyrazolyl)ethyl ether ligands reported by Lin et al [34]., the first example of a three-coordinate copper(I) metallomesogen, have been proposed.

In this work, we report a new class of neutral copper(I) halide complexes (chloride or bromide) with benzoylthiourea (BTU) ligands that combine, in a successfully appealing manner, the 2D supramolecular structures and the liquid crystalline, emission, and 1D ionic conduction properties [35]. This is the first example of anhydrous proton conduction evidenced for a discotic liquid crystal based on a copper(I) metallomesogen provided by the hydrogen-bonding networks formed by NH…X moieties (*X* = halide or oxygen) of the acylthiourea ligand. Nitrogen-based heterocycles are regarded as some of the best candidates for anhydrous proton conduction due to the formation of inter- and intramolecular hydrogen-bonding. Liquid crystals, both pure organic or Pd(II) and Pt(II) metallomesogens, based on related motifs (benzimidazole [36,37], triazole [38], or pyrazole [39]) have been investigated recently. Importantly, the complex nanosegregated organization of columnar liquid crystals can provide 1D nanochannels for ionic transport, in particular proton conduction with a wide range of attractive applications in proton exchange membranes (PEM) [40] or other electro-optical and energy-related devices [41,42,43].

## 2. Results and Discussions

### 2.1. Synthesis and Structural Characterization

**Scheme 1:**
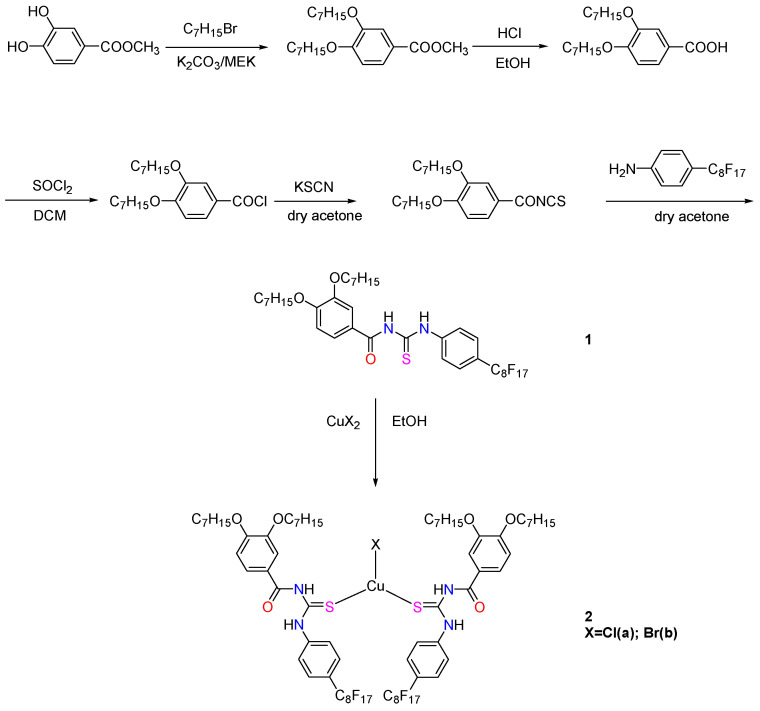
Synthesis of the benzoylthiourea ligand BTU (**1**) and of the copper(I) complexes (**Cu2a** and **Cu2b**).

The synthetic pathway used to prepare the benzoylthiourea ligand **1** (BTU) and its two copper halide complexes, **Cu2a** and **Cu2b**, is depicted in Scheme 1.

The novel benzoylthiourea ligand has, at one side, two heptyloxy chains and, at the other side, a perfluorooctyl group; it was prepared by treating the 3,4-diheptyloxybenzoic acid with thionyl chloride in freshly distilled dichloromethane to give the corresponding acyl chloride. Without any further purification, the acid chloride was treated with potassium thiocyanate in dry acetone to provide the isothiocyanate intermediate that was reacted with 4-perflourooctyloxyaniline to yield the benzoylthiourea compound as a white crystalline solid. The synthetic protocol is based on the methods already described in the literature [44,45,46]. The reaction between metal salts and these versatile *N*-acylthiourea derivatives provides a variety of metal complexes, including both homoleptic and heteroleptic complexes. These ligands can act as a bidentate ligand in the monoanionic form through the O and S atoms or as a monodentate ligand via the thione S atom in the neutral form [47,48,49,50,51,52,53,54,55,56,57,58,59]. 

It is well documented that the complex redox reaction of *N*-acylthiourea derivatives and copper(II) salts produce mono-, di-, or polynuclear species with halide or *S*-thioureato-bridges in which the copper(II) ion is reduced to copper(I). The geometry around the copper(I) could be either plan-trigonal for three-coordinate species or tetrahedral for tetra-coordinate species, proving the versatility of these organic compounds and the coordination flexibility of the copper(I) ion [60,61,62,63,64,65,66]. For example, there is a growing number of reported three-coordinate copper(I) complexes with acylthiourea ligands in which the planar trigonal geometry with a typical butterfly-like structure is stabilized by hydrogen bonding involving the halide ligand, the carbonyl, and the NH groups [67,68,69,70,71]. The same stereochemistry was seen for organosulfur-copper(I) complexes with *S*-coordinated thiosemicarbazone [72] or thione [73]. These three-coordinate copper(I) complexes have two molecules of acylthiourea ligands, coordinated through the thiocarbonyl sulfur atom and one halide ion, yielding an approximately overall planar shape which, in principle, could easily lead to columnar stacking in the liquid crystal phases [74]. However, dimerization with a sulfur-bridged thiourea ligand can occur, yielding dinuclear tetracoordinated copper(I) complexes [60]. In our case, the results of the elemental analysis gave the CuL_2_X stoichiometry (L = *N*-benzoylthiourea ligand). We sought additional support for the nuclearity of the copper(I) complexes from mass spectrometry. Indeed, the electrospray ionisation mass spectrometry (ESI–MS) patterns of the two complexes are similar and show the highest *m*/*z* values at 1867.59 for **Cu2a** and 1867.54 for **Cu2b,** assigned to [Cu+2L]^+^ (calc. 1867.41) and at 901.39 for [L−H]^+^ (calc. 901.23) (Figure S10, Supplementary Materials). 

**Figure 1:**
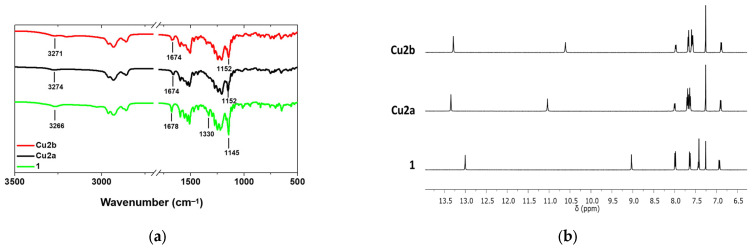
IR spectra (**a**) and the low field region of the ^1^H-NMR spectra (**b**) of the benzoylthiourea ligand 1 and its copper(I) complexes (**Cu2a** and **Cu2b**).

The molecular structure of the two copper(I) complexes were further confirmed based on the elemental analysis results, IR, UV–VIS, and NMR spectroscopy data. The coordination through the C=O groups is ruled out as the position and intensity of the corresponding stretching frequency located at 1678 cm^−1^ in the IR spectrum of free ligand **1** are essentially unchanged, found at 1674 cm^−1^, in the IR spectra of the copper(I) complexes **Cu2a** and **Cu2b** (Figure 1a). The coordination of the copper(I) ion to the sulfur atom of the C=S group is confirmed by the absence in the IR spectra of complexes [71] of the medium intense band assigned to v_C=S_ + ν_C-N_, located at 1330 cm^−1^ in the IR spectrum of uncoordinated ligand **1**, and by the shift to higher wavenumbers, from 1145 cm^−1^ for **1** to 1152cm^−1^ for **Cu2a** and **Cu2b** of the ν_C-N_ frequency. In addition, the apparently decrease in ν_C=O_ frequencies for the ligand and the two copper(I) complexes, compared with the ordinary carbonyl absorption (1700 cm^−1^), could be indicative of the possible formation of intramolecular hydrogen bonding with the N–H groups [75].

The ^1^H and ^13^C-NMR spectra of copper(I) complexes, recorded in CDCl_3_ solvent, support the coordination of the benzoylthiourea ligand to the copper(I) ion. The signals assigned to the two NH singlets are significantly downfield shifted as a consequence of the coordination of the ligand to the copper(I) metal center. A more pronounced shift was observed for the signal assigned to the NH group located between the carbonyl and thiocarbonyl groups of the BTU ligand that is supposed to be involved in the hydrogen bonding with the halide ions (Figure 1b).

### 2.2. Characterization of Liquid Crystalline Properties

#### 2.2.1. DSC and POM Studies

The mesomorphic behaviours of the ligand and the two copper(I) complexes were studied by differential scanning calorimetry (DSC) and polarizing optical microscopy (POM), while the mesophase type was assigned based on powder X-ray diffraction (XRD) studies. The thermal stability of the new compounds was investigated by thermogravimetric analysis (TG). The resulting thermal parameters are presented in Table 1.

**Figure 2:**
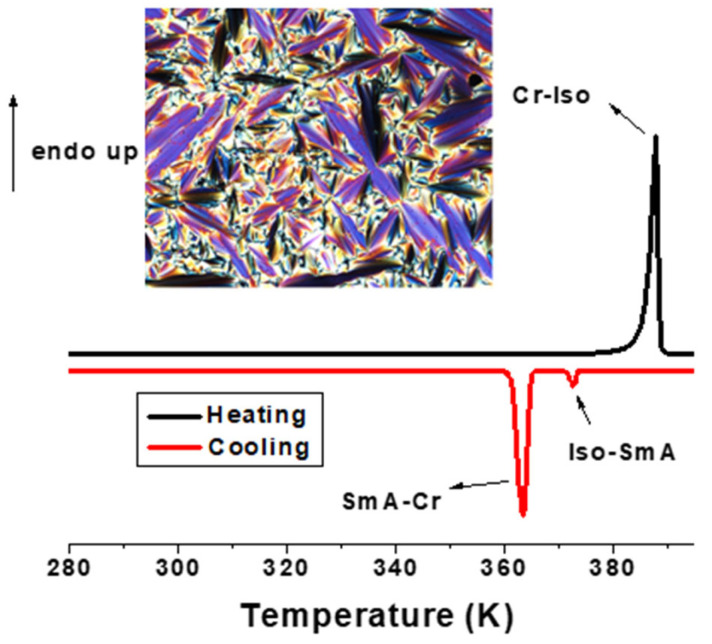
First DSC heating–cooling cycle for ligand **1** (inset: the polarized optical micrograph taken at 370 K on cooling from the isotropic liquid).

In the first heating run, the DSC thermogram of the ligand shows only one thermal event corresponding to the melting process, from a crystalline phase straight to the isotropic state. However, during the cooling run, the two exothermic peaks indicate two different transitions: at a higher temperature, the isotropic to liquid crystal phase transition, followed by a second one, at 365 K, corresponding to the crystallization process (Figure 2). These two transitions are reproducible at further heating–cooling cycles. The POM observations suggested that the monotropic liquid crystalline phase of the ligand could easily be assigned to an SmA phase based on the characteristic fan shape texture accompanied by the homeotropic regions (Figure 2). The related *N*-acyl thiourea compounds functionalized with terminal alkoxy groups on both sides display interesting liquid crystals properties, where the position and length of the alkyl chains have a significant impact on the mesophase type (either nematic or smectic A and C phases) and the corresponding thermal range. The previous studies on related partially fluorinated *N*-acyl thiourea compounds indicated a similar mesomorphic behavior, with an increased stability of lamellar phases and higher transition temperatures, a typical feature of liquid crystals possessing semi- or perfluoro-alkylated chains [76,77], explained by the incompatibility between the aliphatic and perfluoro-alkylated chains, as well as the rigidity of the latter [28,78,79,80,81,82,83,84,85].

**Figure 3:**
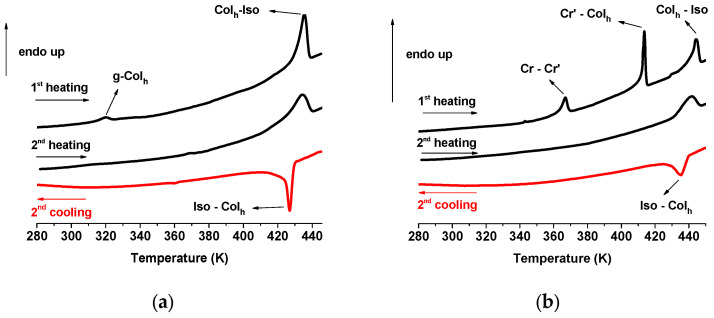
DSC thermograms of the first and second heating–cooling cycles for complex **Cu2a** (**a**) and for complex **Cu2b** (**b**) (10 K·min^−1^).

The thermal behaviors of the two copper(I) complexes are very different in the first heating run. The DSC thermogram of the chloro-complex **Cu2a** shows a glass transition at 318 K followed by a clearing transition at 430 K during the first heating run (Figure 3a). On the contrary, the bromo-complex **Cu2b** is a crystalline solid that, during the first heating run, displays one phase transition between two different crystalline states at 363 K, and the melting to liquid crystal phase at 412 K, followed by the isotropization process at 441 K (Figure 3b). The clearing temperatures are related to the size of the halide ion and, obviously, the clearing temperature of the bromo-complex is slightly higher than the one of the chloro-complex, as expected. The DSC thermograms of both copper(I) complexes show that, on cooling the isotropic liquid, a strong phase transition to the liquid crystal phase occurs, as indicated by the birefringent texture observed by POM. Further cooling did not indicate any crystallization transformations and only a glass transition could be detected at 318 K for **Cu2a** and 313 K for **Cu2b** (temperature recorded at half inflexion point). The following heating–cooling cycles displayed the same two-phase transitions, one corresponding to the isotropic to Col_h_ phase and the glass transition in the same interval found in the first cooling run, meaning that the two copper(I) complexes remain in glassy states at an ambient temperature.

**Figure 4:**
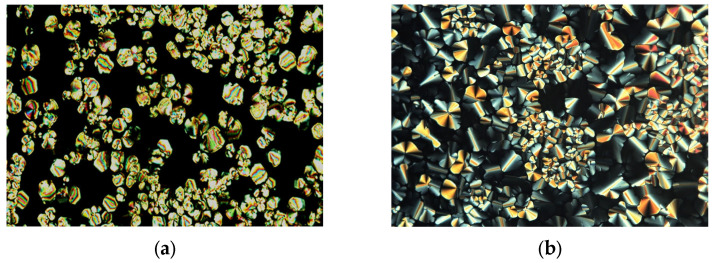

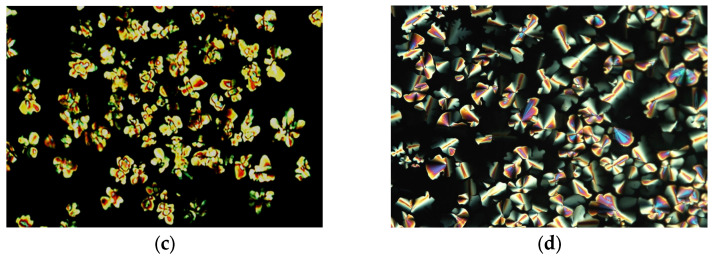
POM micrographs for complex **Cu2a** at 425K (**a**) and 405K (**b**); textures of complex **Cu2b** at 435K (**c**) and 415K (**d**).

The POM observations support this behavior as the texture developed from the isotropic state remains virtually the same after cooling to room temperature when the compounds solidify as a glass (Figure 4).

#### 2.2.2. Thermal Decomposition

**Figure 5:**
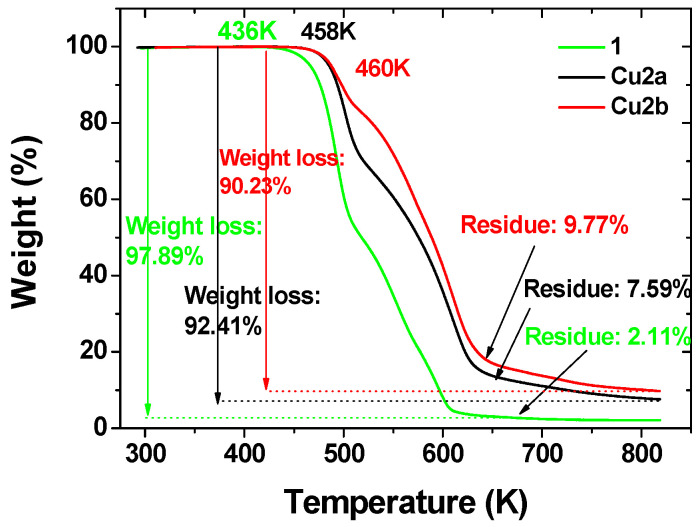
The TG curves for the benzoylthiourea ligand and its copper(I) complexes.

The thermal stability of the BTU ligand and the new copper(I) complexes was checked by thermogravimetric analysis. The samples were heated in a nitrogen flow with a heating rate of 10 K.min^−1^ in the 298–823 K temperature range. The TG curves are shown in Figure 5.

There is no weight loss recorded in the 300–400 K thermal range of the TG curves, confirming that the copper(I) complexes do not have crystallization water or other solvent molecules. The comparison of the amount of the solid residue, estimated from the TG curves shown in Figure 5 (found to be 7.59% for the chloro-complex **Cu2a** and 9.77% for the bromo-complex **Cu2b**), with the calculated value for Cu_2_S (4.17% for **Cu2a** and 4.08% for **Cu2b**) or for CuX (5.20% for **Cu2a** and 7.36% for **Cu2b**), and metallic copper (3.34% for **Cu2a** and 3.26% for **Cu2b**) denotes that the decomposition of these compounds at 825 K is almost completed and the residue is predominantly formed by CuX. The decomposition at high temperatures (above 1100 K) in the nitrogen atmosphere of the copper(I) complexes with sulfur containing ligands can lead to residues consisting of Cu_2_S, CuX, Cu, or a mixture of these products, as shown by XRD investigations [86,87]. Both copper(I) complexes showed an improved thermal stability in comparison with the uncoordinated ligand **1**, with higher decomposition temperatures, 458 K for **Cu2a** and 460 K for **Cu2b**, respectively (Figure 5), that can be accounted to the intramolecular hydrogen bonding involving Cu-X…H-N interactions [88]. The relatively high decomposition temperatures recorded for the two copper(I) complexes have made accessible the variable-temperature XRD measurements that can be used to confirm the nature of the mesophase.

#### 2.2.3. X-ray Diffraction Investigation

**Figure 6:**
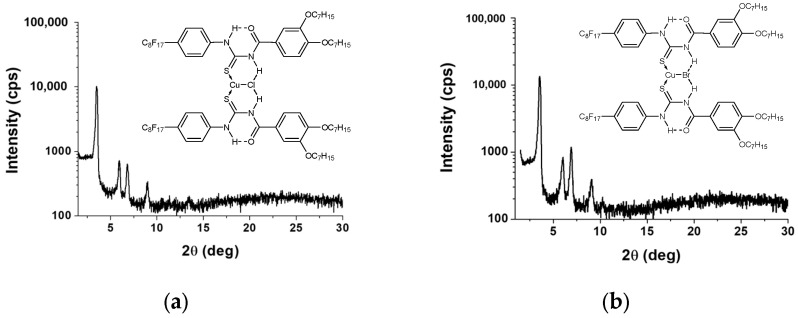
Powder XRD pattern for complex **Cu2a** (**a**) and **Cu2b** (**b**) in the hexagonal columnar mesophase (Col_h_) at 388 K.

In order to avoid the partial decomposition of the copper(I) complexes during the XRD measurements, the samples were cast on a glass slide as dichloromethane solutions and heated with 10K^.^min^−1^ to the temperature of the mesophase existence, but below isotropization temperatures. The XRD diffractograms of **Cu2a** and **Cu2b** recorded at 388K are presented in Figure 6. Clear typical patterns that are characteristic of a hexagonal packing were measured for both complexes. On cooling the samples from the isotropic phase down to the mesomorphic domain, the diffractograms of **Cu2a** and **Cu2b** displayed a strong diffraction peak from the (100) reflection, followed by a series of four weaker sharp peaks with a d-spacing ratio of d/$\sqrt{3}$, d/$\sqrt{4}$, d/$\sqrt{7}$, and d/$\sqrt{9}$, attributed to (110), (200), (210), and (300) reflections, respectively, as depicted in Figure 6 and Figure S13 (Supplementary Materials). The XRD data are indicated in Table 2. In addition to these reflections, another broad and asymmetric peak was observed in the wide-angle region (20–25 degree) in the XRD patterns of the two copper(I) complexes; this could be assigned to the combined lateral interactions of the alkyl chains and π–π stacking interactions. As a consequence of the coordination of the *N*-acylthiourea ligand to the metal center, the fluorophobic effect was reduced, thus preventing the segregation of the fluorocarbon and hydrocarbon chains. The XRD diffractograms of the two copper(I) complexes did not have any broad signal around 5.5 Å that could be assigned to the lateral interactions of the fluorocarbon chains [89,90].

From the two-dimensional hexagonal parameters (d100) calculated as 24.94 Å for **Cu2a** and 25.52 Å for **Cu2b**, the distance between the neighbouring columns were evaluated as 28.80 Å for **Cu2a** and 29.48 Å for **Cu2b**. These two hexagonal lattice parameters are very similar for the two copper(I) complexes, providing the conclusion of an identical packing model in the mesophase. In order to propose a self-assembled packing model, the number of molecules (N) within a volume fraction of thickness h of a column can be calculated with the following relation: hS = NV_mol_, where S is the columnar cross-section, h is the stacking periodicity along the columns of the hexagonal lattice, and V_mol_ is the molecular volume. The V_mol_ can be estimated with the formula V_mol_ = M/ρ·0.6022, where M is the molecular weight and ρ is the density and can be approximated as 1g·cm^−3^. These relationships give N values of 0.82 and 0.84 for **Cu2a** and **Cu2b**, respectively, when h = 3.6 Å (the typical distance for π–π interactions), but 1.02 and 1.04, respectively, when h = 4.5 Å. The stacking distance of 4.5 Å points to the formation of a similar molecular arrangement for the two copper(I) complexes where one molecule is found in the slice of the columns.

### 2.3. UV–VIS and Emission Properties

The photophysical properties of copper(I) complexes have been investigated both in solution and in a solid state at an ambient temperature and the results are summarized in Table 3 [91,92].

**Figure 7:**
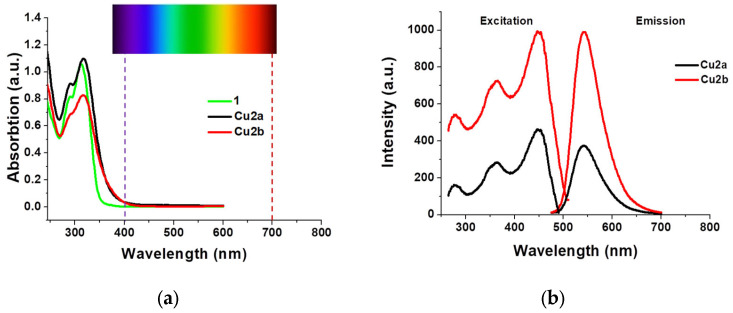
The UV–VIS spectra recorded in dichloromethane solution of ligand 1 (4 × 10^−5^ M), and copper (I) complexes **Cu2a** and **Cu2b** (2 × 10^−5^ M) at ambient temperature (**a**); the excitation and emission spectra of copper(I) complexes **Cu2a** and **Cu2b** recorded in solid state at room temperature (**b**).

The absorption spectra of copper(I) complexes in dichloromethane (Figure 7a) display one highly intense absorption band at ~315 nm with a shoulder at ~290 nm (ε = 34,400–54,900 M^−1^ cm^−1^) that was assigned to the π–π* ligand-centred (LC) transition of the coordinated BTU ligand, based on a similarity with the absorption spectrum of the uncoordinated ligand. This absorption band is slightly red-shifted compared with their position in the electronic spectrum of the free ligand as a consequence of the rigidity of the molecule, resulting from the coordination of the *N*-acylthiourea compound to the metal center. No additional distinct absorption bands have been detected in the electronic spectra of these two complexes but the long tail of the absorption at ~350 nm could be attributed to the characteristic metal to ligand charge transfer (MLCT) and intraligand charge transfer (ILCT) transitions specific to the copper(I) complexes [93,94].

**Figure 8:**
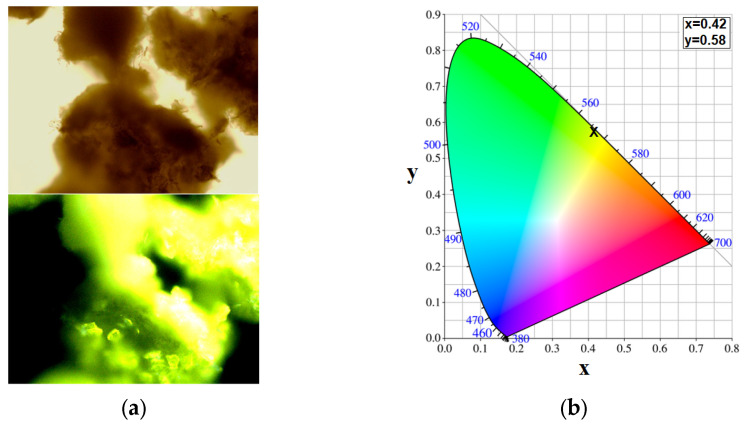
The optical microscopy images of **Cu2b** in normal light and under UV irradiation in 320–360 nm region (**a**) and CIE 1931 (x,y) chromaticity diagram for copper (I) complexes derived from the solid-state emission spectra (**b**).

The ligand and the two copper complexes were almost non-emissive in dichloromethane solution. In addition, the emission of the ligand was not detected in a solid state. The solid-state emission spectra recorded at room temperature for the copper(I) complexes **Cu2a** and **Cu2b** are shown in Figure 7b. The emission spectra in a solid state of the two copper(I) complexes are similar and show one maxima at λ_max_ around 545 nm with a shoulder around 580 nm when the samples are irradiated with λ_exc_ = 365 nm; the color impression analysis for the green–yellow luminescence indicated that x = 0.42 and y = 0.58 (Figure 8b). The green–yellow emission was also visually detected by the optical microscope when the samples were irradiated in the 320–360 nm region (Figure 8a). The photoluminescence at 545 nm and the corresponding excitation peaks above 300 nm (located at 350 and 450 nm) were due to the electronic transitions of the copper(I) complexes’ excited state ^3^MLCT (low energy LE emission) [95]. By heating the two complexes, the photoluminescence intensity was dramatically reduced upon heating from the crystalline state to the mesophase due to the inherent aggregation–caused quenching (ACQ) effect often seen in common luminescent metallomesogens [96]. The highest quantum yield was measured for **Cu2b** and its value of 8% is comparable to other copper(I) complexes with related acyl-thiourea ligands [16]. 

### 2.4. Dielectric Spectroscopy

#### 2.4.1. Electrical Conductivity

**Figure 9:**
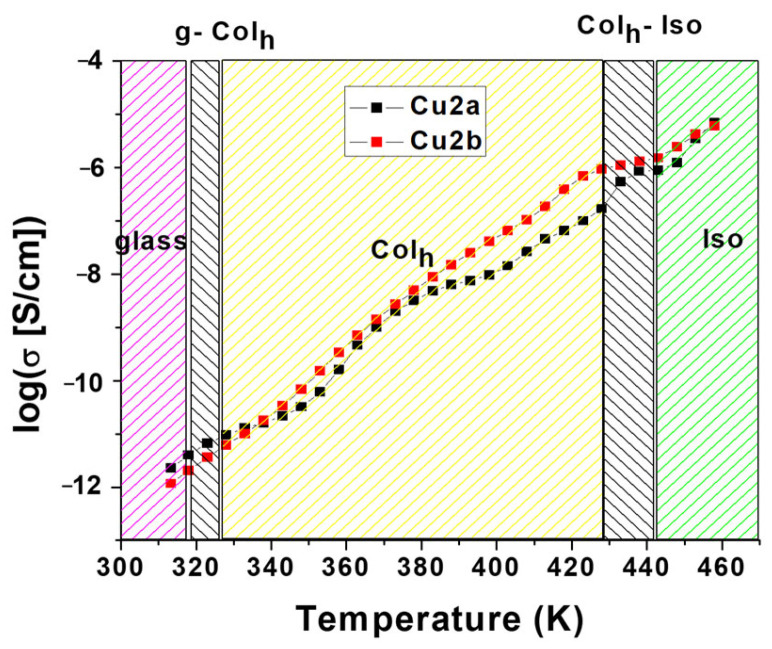
Variation of the electrical conductivity with temperature for the copper(I) complexes **Cu2a** (black squares) and **Cu2b** (red squares), $\sigma =\sigma \left(T\right)$, f = 10 Hz.

The electrical conductivity temperature dependencies, $\sigma =\sigma \left(T\right)$, for the two copper(I) complexes, **Cu2a** and **Cu2b**, recorded for a fixed frequency, f = 10 Hz, are presented in Figure 9.

While the values of the electrical conductivity recorded for the bromo-complex **Cu2b** are slightly higher in comparison with the values recorded for the chloro-complex **Cu2a,** over almost the entire temperature range, the electrical conductivity temperature variation of the two complexes has some similarities. Firstly, it is important to remark on the very large variation of conductivity with temperature, from 10^−12^ S.cm^−1^ to 10^−6^ S.cm^−1^, which is seven orders of magnitude (Table 4).

Then, the growth rate has a “tortuous” variation, up to the temperature 428 K. The electrical conductivity of **Cu2a** presents a jump (a sudden variation) when the temperature rises from 428 K to 432 K, which is the temperature range corresponding to the isotropization process. Furthermore, both conductivities show a stagnation on the ranges 432–443 K and 425 K–443 K, respectively, and higher rates at temperatures above T = 443 K, in the isotropic state, were observed (Figure 9). Because of the different conductivity variation rates with temperature, we conclude the presence of several thermally activated conduction mechanisms.

**Figure 10:**
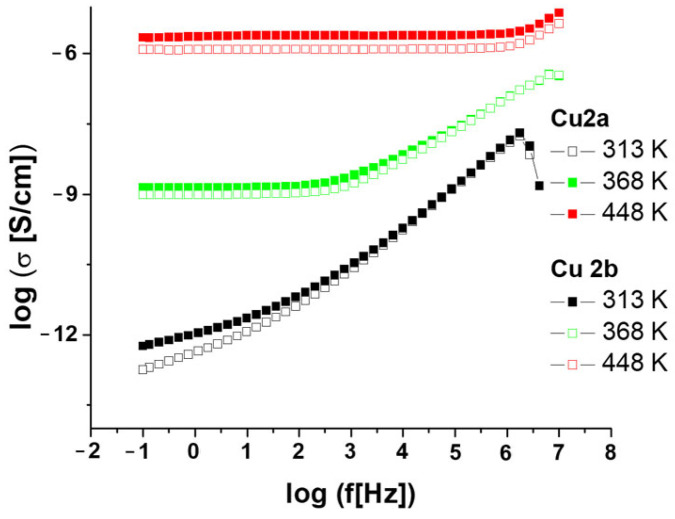
Conductivity spectra versus frequency for copper(I) complexes recorded at different temperatures (313, 368, and 448 K): **Cu2a** (open symbols) and **Cu2b** (solid symbols).

The variation of conductivity with the temperature should be correlated with the conductivity spectra of the two copper complexes presented in Figure 10, which were recorded at different temperatures. As can be seen in Figure 10, the plateau region, where the conductivity is approximately constant as the frequency increases, extends to higher frequencies in the case of higher temperature values. The plateau region can be attributed to the DC conductivity due to the movement of charge carriers by diffusion, and the ascending part to the AC conductivity or/and some dielectric relaxation processes. As a consequence, in the two temperature ranges, the electrical behaviour of the studied complexes has different features: (a) the interval of relatively low temperatures, T = 313–358 K, where the electrical properties are predominantly determined by the dielectric relaxation processes; (b) the interval of relatively high temperatures, 358–453 K, where the electrical properties are predominantly influenced by the electrical conduction.

The important question arises: *what type of conduction characterizes these samples?* To answer this question, we noticed that the presence of free charge carriers has an effect on the permittivity spectra in the low frequency region. Experimentally, it is known that, for a purely ohmic (electronic) conduction, the dielectric losses have a linear increase with the decrease in frequency, but no contribution to the permittivity value appears. At the same time, the real part of conductivity is constant [97]. On the other hand, in the case of ionic conductivity, it is known that the dielectric losses, but also the dielectric constant, increase with the decrease in frequency. In the sense of increasing the frequency from low to high values, the conductivity first presents a flat region followed by an ascending branch where the conductivity increases proportionally to the frequency at a certain power $\sigma \prime \approx \omega^{s}$, 0<s<1.

In the present study, the electrical conductivity spectra, represented in a double logarithmic scale, contains a part with an almost constant value, in the region of low and medium frequencies, continued with a proportional increase with log (*ω*), at higher frequencies; the slope of the line has a sub-unit value (Figure 10). The flat region of the conductivity extends to the higher frequencies as the temperature increases. In the limit f→0(ω→0), the *DC* value of electrical conductivity is obtained, $\sigma_{DC}$ [97,98]. To analyze these spectra, Joncher proposes the following equation regarding the real part of electrical conductivity, σ′ω=Reσ*ω=σDC+σACω, where the alternative current component of the conductivity, $\sigma_{AC}\left(\omega \right)≅A\omega^{s}$, complies with a power law in relation to frequency [98], called the ‘universal dielectric response’. It should be noted that a wide variety of homogeneous or heterogeneous samples with a disordered structure, in solid, liquid, or composite states, show the same kind of dependence of electrical conductivity depending on frequency. Electrical relaxation measurements are commonly used to characterize the dynamics of ionic transport in ionically conducting materials.

The experimental data were fitted with a simple, three-parameter expression for the “universal dielectric response”, as follows [99,100]:

(1) $$\sigma \prime \left(\omega \right)=\sigma_{DC}\left[1+{\left(\omega /\omega_{p}\right)}^{N}\right]$$

where the exponent *N* has a subunitary value, 0≤N<1. Typically, for the ionic conduction, *N* has values in the range 0.6 < *N* < 0.9. The characteristic frequency (studied in terms of angular valocity), $\omega_{p}$, is the frequency from which the alternative current conduction begins to activate, $\sigma_{AC}=\sigma_{DC}$ or $\sigma \prime \left(\omega_{p}\right)=2\sigma_{DC}$. Equation (1) allows the establishment of a correlation between the relaxation (dispersion) of macroscopic electrical conduction and the microscopic movement of ions in materials with ion conduction. *DC* electrical conductivity, $\sigma_{DC}$, and the characteristic frequency, *ω_p_*, are related by the Barton–Nakajima–Namikawa (BNN) equation [101], $\sigma_{DC}=\varepsilon_{0}\varepsilon_{r,\infty }\omega_{p}$, where $\varepsilon_{r,\infty }=\underset{\omega \to \infty }{\lim}\varepsilon_{r}\left(\omega \right)$ is the relative permittivity in the ω→∞ limit. All parameters are temperature dependent: $\sigma_{DC}\left(T\right)$, $\omega_{p}\left(T\right)$, $N\left(T\right)$. The temperature dependence of the exponent *N* provides information about the mechanism involved in the AC electrical conductivity. In most situations, *DC* electrical conduction complies with the Arrhenius law, but there are also exceptions when temperature dependence complies with the empirical law of Vogel–Fulcher–Tamman.

The frequency (pulsation), $\omega_{p}\left(T\right)$, depends on the temperature in a similar way, as does the *DC* conductivity, $\sigma_{DC}$, which supports the BNB equation [101]. 

The variation of the exponent, *N*, with temperature depends on the materials. Thus, for some types of samples, *N* decreases with temperature [102], while for others, *N* increases with temperature [102,103], or it can display a non-monotonous variation, presenting a maximum [104]. By performing the fitting of the experimental conductivity spectra with Equation (3), the calculated values of the *N* parameter were found to be between 0.610 and 0.915 for **Cu2a**, and between 0.536 and 0.862 for **Cu2b**, reinforcing the assignment of the ionic conduction [99]. Additionally, for both complexes, N shows a tendency to increase with temperature. 

For the two copper(I) complexes, the charge carriers are highly mobile ions (e.g., protons, or other small ions). Based on these arguments and considering that the copper(I) complexes have no other possible diffusible ions, the measured electrical conductivity can be assigned to proton conductivity. The hydrogen-bonding networks formed in the copper(I) complexes provide continuous pathways for 1D proton conduction based on the proton transfer in the N-H…X moieties (X = halide or oxygen) of the acylthiourea ligand. It is easily observed that the liquid crystal phases favored anhydrous proton conduction in these copper(I) complexes and a proton conductivity of 2.97 × 10^−7^ S.cm^−1^ was achieved at 430K for **Cu2a** and 1.37 × 10^−6^ for **Cu2b** S.cm^−1^ at 440K (Table 4). For example, similar maximum values of the proton conductivity, in the 10^−5^–10^−9^ S.cm^−1^ range, were reported for palladium(II) complexes with pyridyl or isoquinoline-functionalized pyrazolate ligands in the temperature range of the existence of columnar mesophase [39].

#### 2.4.2. Dielectric Properties

**Figure 11:**
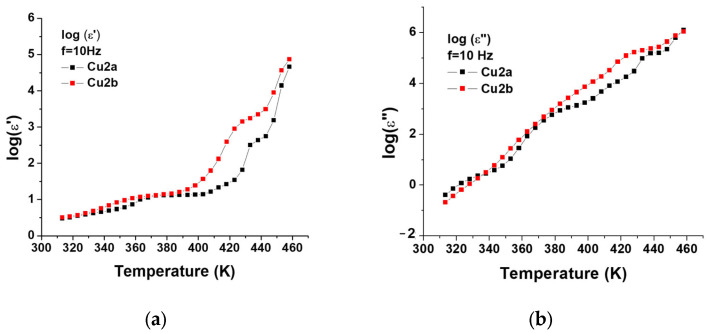
Variation of dielectric constant as a function of temperature (**a**) and the dielectric loss versus temperature (**b**), ε′ = ε′ (T), f = 10 Hz.

The ionic conduction mechanism assigned, based on the variation of electrical conductivity as a function of temperature and frequency, is also supported by the dielectric properties of the two copper(I) complexes. The temperature variation curves of the dielectric constant are presented in Figure 11a and the variation of dielectric loss as a function of temperature, for both copper(I) compounds, at a constant frequency of f = 10 Hz, are presented in Figure 11b.

A similar trend in the variation rate was observed in the case of the dielectric constant and dielectric loss (Figure 11), as found for the electrical conductivity (Figure 9) in the high temperatures domain, over 420 K, where a slight stagnation followed by a faster growth was measured. However, it is worth mentioning here that both the dielectric constant and the dielectric losses have high and very high values up to $5\cdot 10^{04}$ and $1\cdot 10^{06}$, respectively, which supports the conclusion of the existence of an important electrical conductivity, characteristic of these copper(I) complexes.

In order to highlight the specific mechanisms of electrical conductivity and those of dielectric relaxation, the Cole–Cole representation was chosen: dielectric losses as a function of dielectric constant, $\varepsilon \prime \prime =\varepsilon \prime \prime \left(\varepsilon \prime \right)$, in a double logarithmic scale, $\log\varepsilon \prime \prime =f\left(\log\left(\varepsilon \prime \right)\right)$.

**Figure 12:**
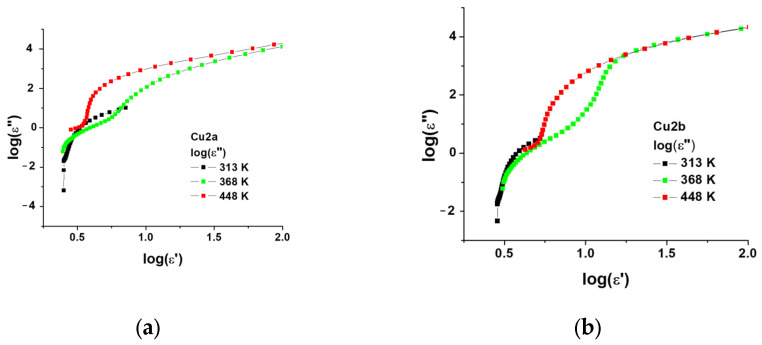
Dielectric losses versus dielectric constant for the two copper(I) complexes at fixed temperatures: T_1_ = 313 K, T_2_ = 368 K, and T_3_ = 448 K: **Cu2a** (**a**) and **Cu2b** (**b**).

The overviews of the dielectric losses versus the dielectric constant for the two copper complexes at different temperatures, presented in Figure S11 (Supplementary Materials), show the whole range of values for the two components of the dielectric permittivity. In order to be able to get further insights, the ranges of values were narrowed, and these enlarged regions are depicted in Figure 12.

At 313 K (solid black squares), an incomplete semicircle is observed, indicating a single dielectric relaxation process, which was assigned to the “alpha” relaxation process. This semicircle shows a pronounced deformation due to the use of the logarithmic scale. At 368 K (solid green squares), two incomplete semicircles were evidenced. The small one corresponds to the dielectric “alpha” relaxation process while the big one corresponds to a dielectric relaxation at a lower frequency, assigned to the “beta” relaxation process. The latter semicircle is extended by an ascending line. Importantly, the rising branch, on the right side of the figure, can be ascribed to the electrical conductivity [97,98]. Finally, at 448.15 K (solid red squares), the “alpha” relaxation process is no longer visible as the “beta” relaxation process predominates. Similar to the results recorded at 368 K, the ascending branch is naturally attributed to electrical conductivity. An important increase, by a few orders of magnitude, of the dielectric constant and of the dielectric losses at low frequencies was evidenced. For temperatures higher than 348 K, the “intensity” of the dielectric relaxation process is artificially amplified by the logarithmic scale used. On a linear scale, these processes would not have been observed. Therefore, the electrical conductivity is the main mechanism that manifests itself at medium and high temperatures. Moreover, the movement of the charge carriers under the effect of the sinusoidal electric field also has a great effect on the values of losses and on the values of the dielectric constant (as can be seen on the rising part of the curve on the right side, Figure 12). 

## 3. Conclusions

New three-coordinate copper(I) complexes with a combination of emission properties in a solid state, 1D ionic conduction properties, and liquid crystalline properties on a long thermal range, from 313 up to 440 K, are reported. The mesophase thermal range can be controlled by the exchange of the halide ion coordinated to a metal center. The complexes lack emission in air-equilibrated solution and in the hexagonal columnar phase at elevated temperatures but show luminescence quantum yields up to 8% in a crystalline state. Considering the very high values of the dielectric constant and of the dielectric losses, up to 5 × 10^4^ and 1 × 10^6^, respectively, the existence of an important electrical conductivity has been attributed to the anhydrous proton conduction supported by the hexagonal columnar organization at higher temperatures of the copper(I) complexes and provided by the hydrogen-bonding networks formed by NH…X moieties (X = halide or oxygen) of the *N*-acylthiourea ligand. The highest value of proton conductivity, 2.97 × 10^−7^ S.cm^−1^, was achieved at 430K for **Cu2a** and 1.37 × 10^−6^ S.cm^−1^ for **Cu2b** S·cm^−1^ at 440 K. 

The design of these copper(I) complexes represents a totally new approach to the feasible preparation of luminescent liquid crystals showing anhydrous proton conduction that opens the way for the development of such materials by grafting different mesogenic groups on related *N*-acylthiourea neutral ligands.

## 4. Experimental Section

### 4.1. Characterization Methods

The chemicals employed in this study were used without further purification as received from the suppliers. The elemental analyses were performed with EuroEA 3300 instrument. The purity of the new compounds was checked by ^1^H-NMR and ^13^C-NMR spectroscopy using a Bruker spectrometer operating at 500 MHz and CDCl_3_ as solvent. ^1^H chemical shifts were referenced to the solvent peak position, *δ* 7.26 ppm. Fourier transformed infrared (FTIR) spectra were measured at room temperature from 4000 to 400 cm^−^^1^ on a Bruker spectrophotometer in KBr discs and the UV–VIS spectra were recorded in dichloromethane solution on a Jasco V650 spectrophotometer. For electrospray ionisation mass spectrometry (ESI–MS) analysis, approximately 10 mg of each complex was dissolved in a mixture of dichloromethane and methanol (8:2) and directly injected into a Velos Pro ion trap mass spectrometer (Thermo Fisher Scientific, Waltham, MA, USA) at a flow rate of 5 μL/min running in ESI + Full MS mode, Spray Voltage: 3 kV, Capillary Temperature: 375 °C, *m*/*z* ranges from 100 to 2000 and 1000 to 2000.

The optical textures of the benzoylthiourea ligand and its two copper(I) complexes were observed by polarizing optical light microscopy (POM) using a Nikon 50iPol microscope equipped with a Linkam THMS600 hot stage and TMS94 control processor. The samples were sandwiched between two untreated glass slides. The DSC (differential scanning calorimetry) experiments were carried out with a Diamond DSC Perkin Elmer instrument at 10°/min scanning rate after being encapsulated in aluminium pans. At least two heating/cooling cycles were performed for each sample. Thermogravimetric analyses for all samples were performed on a TA Q50 TGA instrument using alumina crucibles and nitrogen as purging gas. The samples were heated with 10 °C min^−1^ rate from room temperature to 550 °C. 

The nature of the mesophase was analysed by X-ray diffraction technique. The powder X-ray diffraction measurements were made on a D8 Advance diffractometer (Bruker AXS GmbH, Karlsruhe, Germany) in parallel beam setting, with a monochromatized Cu–Kα1 radiation (λ = 1.5406 Å), a scintillation detector, and a horizontal sample stage. The measurements were performed in symmetric (θ–θ) geometry in the 2θ range from 1.5° to 30° in steps of 0.02°, with measuring times per step in the 5–40 s range. The temperature control of the samples during measurements was achieved by adapting a home-made heating stage to the sample stage of the diffractometer.

The photoluminescence (PL) spectra were recorded at room temperature in solid state and the samples deposited on a glass slide, with an OceanOptics QE65PRO spectrometer attached to the polarizing optical microscope and using a Nikon Intensilight excitation source or a LED light source (LLS-LED, OceanOptics, λ = 365 nm). The photoluminescence, excitation spectra, and photoluminescence decay curves were recorded at room temperature by using a FluoroMax 4P spectrophotometer; for the quantum efficiency, we have used the Quanta–Phi accessory.

The dielectric spectroscopy (DS) measurements were performed using a Broadband Dielectric Spectrometer, Novocontrol, consisting of the Alpha-A High Performance Frequency Analyzer in the LF domain 0.1–10 MHz equipped with WinDETA software. The spectra have been registered in the (313–458) K temperature domain. Temperatures were controlled within 0.2 K. Alternative voltage was set to 0.5 V. 

### 4.2. Preparation of N-(4-Perfluorooctylphenylcarbamothioyl)-3,4-Diheptyloxybenzamide (1)

The preparation of the benzoylthiourea (BTU) ligand **1** is based on the method described earlier [45,46,74]. The 3,4-diheptyloxybenzoic acid (6 mmol) was treated with an excess of thionyl chloride (25 mmol) in freshly distilled dichloromethane (30 mL) for 3 h and heating under reflux. After this period, the excess of thionyl chloride and the solvent were removed under reduced pressure using a rotary evaporator. The corresponding acid chloride was not purified further and it was used as isolated in the next step. Dry acetone (10 mL) was poured over the acid chloride followed by the dropwise addition of a solution of ammonium thiocyanate (6 mmol) in acetone (15 mL), under nitrogen. The resulting mixture was heated under reflux for a period of 30 min. Addition of the NH_4_SCN solution produced a cloudy white precipitate. Further, the mixture was cooled down to room temperature, after which a solution of *p*-perfluoroctylaniline (5.6 mmol) in acetone (15 mL) was added dropwise for a period of 30 min. The reaction mixture was stirred for 2 h and then it was poured in 100 mL of deionized water. The precipitate was filtered off and washed several times with water and ethanol and then recrystallized two times from a mixture of dichloromethane/ethanol to yield a white crystalline solid. 

**Compound 1.** White crystalline solid. Yield: 78%. Anal. Calcd. For C_36_H_39_F_17_N_2_O_3_S (%): C, 47.89; H. 4.32; N, 3.10; Found (%): C, 48.52; H, 4.41; N, 3.44. 

^1^H-NMR (500 MHz, ppm, CDCl_3_) δ 13.00 (s, 1H), 9.03 (s, 1H), 7.99 (d, *J* = 8.4 Hz, 2H), 7.64 (d, *J* = 8.3 Hz, 2H), 7.43 (d, *J* = 8.7 Hz, 2H), 6.93 (d, *J* = 8.2 Hz, 1H), 4.08 (q, *J* = 6.6 Hz, 4H), 1.92–1.80 (m, 4H), 1.54–1.44 (m, 4H), 1.43–1.26 (m, 12H), 0.90 (t, *J* = 6.5 Hz, 6H). 

^13^C-NMR (125 MHz, ppm, CDCl_3_) δ 178.39(s), 166.64(s), 154.31(s), 149.47(s), 141.09(s), 127.67(t, ^3^J_C-F_ = 6.5Hz), 126.52(t, ^2^J_C-F_ = 24.5Hz), 123.23(s), 122.98(s), 120.97(s), 112.52(s), 112.07(s), 69.51(s), 69.22(s), 31.79(s), 31.77(s), 29.13(s), 29.05(s), 29.01(s), 28.99(s), 25.94(s), 25.90(s), 22.60(s), 22.58(s), 14.08(s), 14.07(s). 

IR (KBr, cm^−1^) 3266 (ν**_NH_**), 2961, 2930, 2859 (ν**_C-H_**), 1678 (ν**_C=O_)**; 1509 (ν**_C-N_**), 1330 (ν**_C=S_**_+_ ν**_C-N_**), 1276 (ν**_Ar-O_)** 1249 (ν**_C-N_**), 1145 (ν**_C-N_**).

### 4.3. Preparation of Copper(I) Complexes (**Cu2a** and **Cu2b**)

The corresponding BTU ligand (1 mmol) was dissolved in hot ethanol (10 mL). Separately, a solution of the corresponding halide copper(II) salt (CuX_2_, *X* = Cl, or Br, 0.5 mmol) in hot ethanol (5 mL) was prepared. The two solutions were slowly mixed together over a period of 10 min. The reaction mixture was stirred and heated under reflux for 1.5 h when a green–yellow precipitate was formed in each case. The resulting precipitate was filtered off while the mixture was hot and then washed several times with ethanol and dried in vacuum. The yields were calculated based on copper salts. 

**Compound Cu2a.** Yellow-green solid. Yield: 78%. Anal. Calcd. For C_72_H_78_F_34_N_4_O_6_S_2_CuCl (%): C, 45.41; H, 4.13; N, 2.94; Found (%): C, 44.57; H, 4.61; N, 3.14. 

^1^H NMR (500 MHz, ppm, CDCl_3_) δ 13.34 (s, 1H), 11.04 (s, 1H), 8.00 (d, *J* = 8.4 Hz, 1H), 7.75–7.58 (m, 5H), 6.90 (d, *J* = 8.5 Hz, 1H), 4.09 (t, *J* = 6.5 Hz, 2H), 4.04 (t, *J* = 6.5 Hz, 2H), 1.89–1.81 (m, 2H), 1.80–1.71 (m, 2H), 1.52–1.20 (m, 16H), 0.95–0.84 (m, *J* = 15.7, 7.0 Hz, 6H).

^13^C NMR (126 MHz, ppm, CDCl_3_) δ 179.23(s), 168.99(s), 154.60(s), 148.95(s), 139.94(s), 127.86(m, ^2^J_C-F_ = 24.5, ^3^J_C-F_ = 6.5Hz), 125.36(s), 123.88(s), 122.24(s), 113.43(s), 111.72(s), 69.49(s), 69.06(s), 31.93(s), 31.81(s), 31.79(s), 29.70(s), 29.67(s), 29.37(s), 29.18(s), 29.11(s), 29.07(s), 25.98(s), 25.94(s), 22.62(s), 22.60(s), 14.09(s), 14.07(s).

IR (KBr, cm^−1^) 3274 (ν**_NH_**), 2960, 2932, 2859 (ν**_C-H_**), 1674 (ν**_C=O_)**; 1512(ν**_C-N_**), 1275 (ν**_Ar-O_)** 1244 (ν**_C-N_**), 1152 (ν**_C-N_**).

**Compound Cu2b.** Yellow-green solid. Yield: 97%. Anal. Calcd. For C_72_H_78_F_34_N_4_O_6_S_2_CuBr (%): C, 44.37; H, 4.03; N, 2.87; Found (%): C, 44.45; H, 3.48; N, 3.04. 

^1^H NMR (500 MHz, ppm, CDCl_3_) δ 13.29 (s, 1H), 10.61 (s, 1H), 7.97 (d, *J* = 8.4 Hz, 1H), 7.67 (m, *J* = 8.3 Hz, 2H), 7.61–7.55 (m, 3H), 6.89 (d, *J* = 8.5 Hz, 1H), 4.09 (t, *J* = 6.4 Hz, 2H), 3.99 (t, *J* = 6.4 Hz, 2H), 1.93–1.68 (m, 4H), 1.50–1.16 (m, 16H), 0.98-0.78 (m, *J* = 15.2, 7.0 Hz, 6H).

^13^C NMR (126 MHz, ppm, CDCl_3_) δ 178.04(s), 168.65(s), 154.69(s), 148.99(s), 139.79(s),127.84(m, ^2^J_C-F_ = 24.5, ^3^J_C-F_ = 6.5Hz), 125.15(s), 123.89(s), 121.93(s), 113.26(s), 111.69(s), 69.54(s), 69.04(s), 31.80(s), 31.77(s), 29.70(s), 29.19(s), 29.11(s), 29.05(s), 29.02(s), 25.99(s), 25.92(s), 22.60(s), 22.58(s), 14.06(s), 14.04(s). 

IR (KBr, cm^−1^) 3271 (ν**_NH_**), 2960, 2931, 2860 (ν**_C-H_**), 1674 (ν**_C=O_)**; 1506(ν**_C-N_**), 1276 (ν**_Ar-O_)** 1244 (ν**_C-N_**), 1152 (ν**_C-N_**).

## Figures and Tables

**Table 1:** Thermal parameters for the BTU ligand and its copper(I) complexes.

Compound	Transitions (T/K, ∆H/kJmol^−1^) ^1,2^
**1 (BTU)**	1st: Cr 386 (45.1) Iso 373 (2.1) SmA 365 (33.2) Cr 2nd: Cr 385 (45.0) Iso 373 (2.0) SmA 364 (32.9) Cr
**Cu2a**	1st: g 318 Col_h_ 430 (22.9) Iso 429 (23.0) Col_h_ 318 g 2nd: g 318 Col_h_ 429 (22.8) Iso 428 (23.0) Col_h_ 318 g
**Cu2b**	1st: Cr 363 (7.9) Cr’ 412 (18.0) Col_h_ 441 (16.7) Iso 440 (16.9) Col_h_ 313 g 2nd: g 313 Col_h_ (17.1) 440 Iso 439 (17.2) Col_h_ 313 g

^1^ The corresponding transition temperatures and enthalpies are given in parenthesis; Cr and Cr-crystalline phases, g—glassy state, Iso—isotropic phase, SmA—smectic A phase, Col_h_ —hexagonal columnar phase; ^2^ Data recorded in the first and second heating runs.

**Table 2:** XRD data for copper(I) complexes.

Compound	Mesophase	T(K)	Indexation	d-Spacing	d-Spacing	Lattice
				exp.(Å)	calc. (Å)	parameter (Å) ^a^
**Cu2a**	Col_h_	388	100	24.94	24.94	a = 28.80
			110	14.24	14.40	
			200	12.34	12.47	
			210	9.28	9.43	
			300	8.22	8.31	
**Cu2b**	Col_h_	388	100	25.52	25.52	a = 29.48
			110	14.67	14.73	
			200	12.62	12.76	
			210	9.52	9.64	
			300	8.42	8.51	

^a^ the Col_h_ lattice parameter a = 2<d_100_>/$\sqrt{3}$.

**Table 3:** Absorption data in dichloromethane solution and emission data in solid state for the BTU ligand and copper(I) complexes.

Compound	Absorption, λ_max_/nm (ε × 10^−3^/M^−1^ cm^−1^)	Emission		
		Solid λ_em_/nm (λ_exc_ = 365nm)	τ/μs	Φ (%)
**1**	290 (20.4), 313 (26.4)	-	-	-
**Cu2a**	291 (45.7), 317 (54.9)	545, 580 (sh)	11, 172	2.5
**Cu2b**	290 (sh, 34.4), 316 (41.4)	546, 580 (sh)	9, 176	8.0

**Table 4:** Ionic conductivities and activation energies in the solid state (glassy state, 320 K) mesophase (370 and 400 K), and isotropic liquids (450 K) for complexes **Cu2a** and **Cu2b**. The calculation of the activation energy E_A_ is presented in SI.

Compound	Conductivity (σ/S·cm^−1^)				E_A_ (Mesophase)
	Glass	Mesophase		Isotropic	
	320 K	370 K	400 K	450 K	(eV)
**Cu2a**	5.05 × 10^−12^	1.32 × 10^−9^	1.12 × 10^−8^	1.88 × 10^−6^	1.2
**Cu2b**	2.64 × 10^−12^	1.86 × 10^−9^	4.98 × 10^−8^	3.03 × 10^−6^	1.5

## Data Availability

Not applicable.

## Supplementary Materials

The following supporting information can be downloaded at: https://www.mdpi.com/article/10.3390/molecules28104196/s1, Figure S1: IR spectrum of compound 1; Figure S2. IR spectrum of compound **Cu2a**; Figure S3: IR spectrum of compound **Cu2b**; Figure S4. ^1^H-NMR spectrum of ligand **1**; Figure S5: ^13^C-NMR spectrum of ligand **1**; Figure S6: ^1^H-NMR spectrum of complex **Cu2a**; Figure S7: ^13^C-NMR spectrum of complex **Cu2a**; Figure S8: ^1^H-NMR spectrum of complex **Cu2b**; Figure S9: ^13^C-NMR spectrum of complex **Cu2b**; Figure S10: Mass spectrometry identification of [Cu+2L]^+^ and [L-H]^+^ ions of **Cu2a** (A) and **Cu2b** (B) corresponding to calculated m/z; Figure S11: DC conductivity (natural logarithm) versus 1000/T and the linear fitting function for **Cu2b**; Figure S12: Overviews: dielectric losses versus dielectric constant for the coppper(I) complexes at different temperatures (T1 = 313.15 K, T2 = 368.15 K and T3 = 448 K): **Cu2a** (a) and **Cu2b** (b); Figure S13: Powder XRD pattern for complex **Cu2a** (a) and **Cu2b** (b) in the hexagonal columnar mesophase (Col_h_) at 388 K recorded on heating; Figure S14. Photoluminescence decay profiles for **Cu1a** (a) and **Cu2b** (b) represented in the log scale, showing the two exponential decays.

## Sample Availability

Samples of the compounds are available from the authors.
