# Supplementary material for: Columnar Liquid Crystals of Copper(I) Complexes with Ionic Conductivity and Solid State Emission
Source: Molecules. 2023 May 19;28(10):4196. doi: 10.3390/molecules28104196 (PMC10220757; doi:10.3390/molecules28104196)
Supplement: Supplementary file 1 [file molecules-28-04196-s001.zip › molecules-2396650-supplementary.pdf]

Supplementary material

Columnar liquid crystals of copper(I) complexes with ionic conductivity and solid state emission

Viorel Cîrcu[1,*], Constantin P. Ganea[2], Mihail Secu[2], Doina Manaila Maximean[3,4*], George Cătălin Marinescu[5], Roua Gabriela Popescu[5,6], Iuliana Pasuk[2]

[1]Department of Inorganic and Organic Chemistry, Biochemistry and Catalysis, University of Bucharest, 4-12 Regina Elisabeta Bld., sector 5, Bucharest 030018, Romania
[2]National Institute of Materials Physics, Atomistilor 405A, 077125 Magurele, Romania
[3] University Politehnica of Bucharest, Department of Physics, 313 Spl. Independentei, 060042, Bucharest, Romania
[4] Academy of Romanian Scientists, 3 Ilfov str., RO-050094, Bucharest, Romania
[5]Asociația Independent Research, 58 Timișului, sector 1, Bucharest 012416, Romania
[6]Department of Biochemistry and Molecular Biology, University of Bucharest, 91-95 Spl. Independentei, 050095 Bucharest, Romania.

Corresponding authors: viorel.circu@chimie.unibuc.ro, manaila@physics.pub.ro

Content:

1. **Figure S1.** IR spectrum of compound **1**. p. 2
2. **Figure S2.** IR spectrum of compound **Cu2a**. p. 2
3. **Figure S3.** IR spectrum of compound **Cu2b**. p. 2
4. **Figure S4.** ^{1}H-NMR spectrum of ligand **1**. p. 3
5. **Figure S5.** ^{13}C-NMR spectrum of ligand **1**. p. 3
6. **Figure S6.** ^{1}H-NMR spectrum of complex **Cu2a**. p. 4
7. **Figure S7.** ^{13}C-NMR spectrum of complex **Cu2a**. p. 4
8. **Figure S8.** ^{1}H-NMR spectrum of complex **Cu2b**. p. 5
9. **Figure S9.** ^{13}C-NMR spectrum of complex **Cu2b**. p. 5
10. **Figure S10**. Mass spectrometry identification of $[Cu+2L]^+$ and $[L-H]^+$ ions of **Cu2a** (A) and **Cu2b** (B) corresponding to calculated m/z. p. 6
11. **Figure S11.** DC conductivity (natural logarithm) versus 1000/T and the linear fitting function for **Cu2b**. p. 7
12. **Figure S12**. Overviews: dielectric losses versus dielectric constant for the coppper(I) complexes at different temperatures (T_1=313.15 K, T_2=368.15 K and T_3=448 K): **Cu2a** (a) and **Cu2b** (b). p. 8

Figure S13. Powder XRD pattern for complex **Cu2a** (a) and **Cu2b** (b) in the hexagonal columnar mesophase (Col_h) at 388 K recorded on heating. p. 8

Figure S14. Photoluminescence decay profiles for **Cu1a** (a) and **Cu2b** (b) represented in the log scale, showing the two exponential decays. p. 9

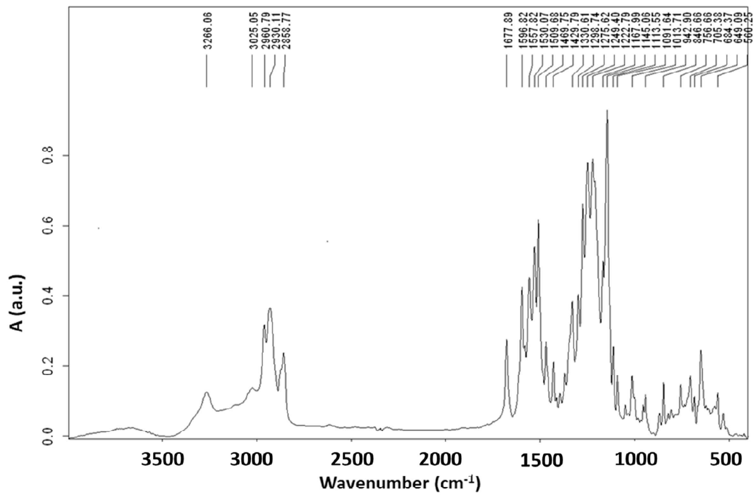

Figure S1. IR spectrum of compound **1**.

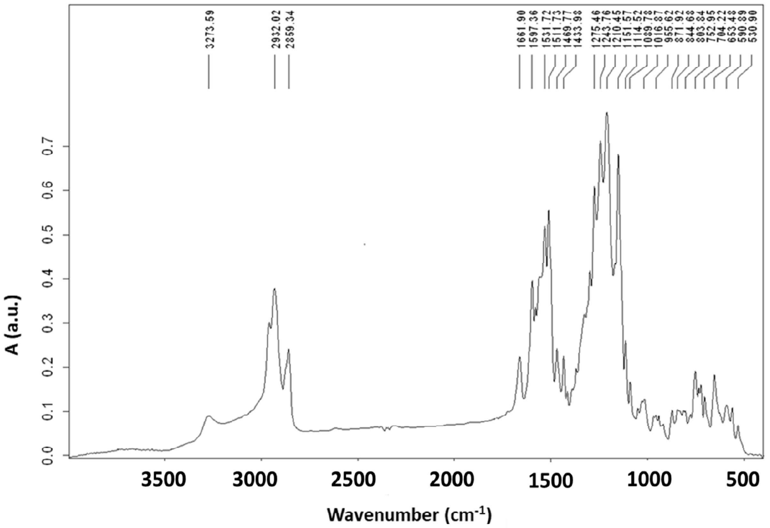

Figure S2. IR spectrum of compound **Cu2a**.

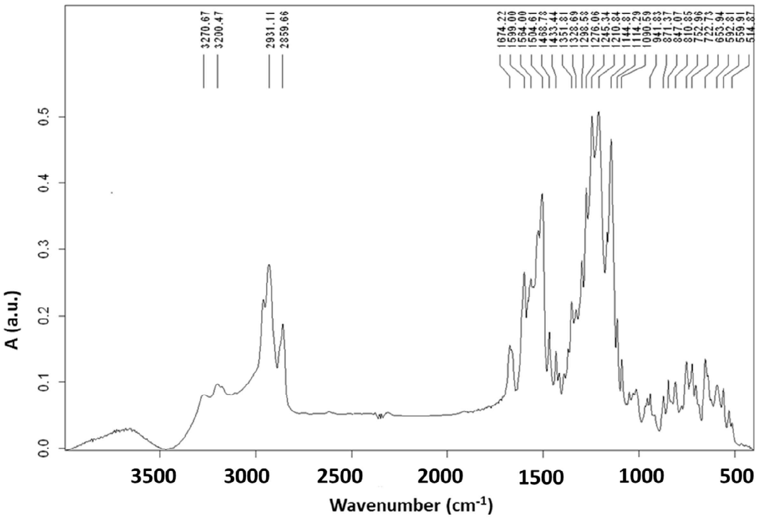

Figure S3. IR spectrum of compound **Cu2b**.

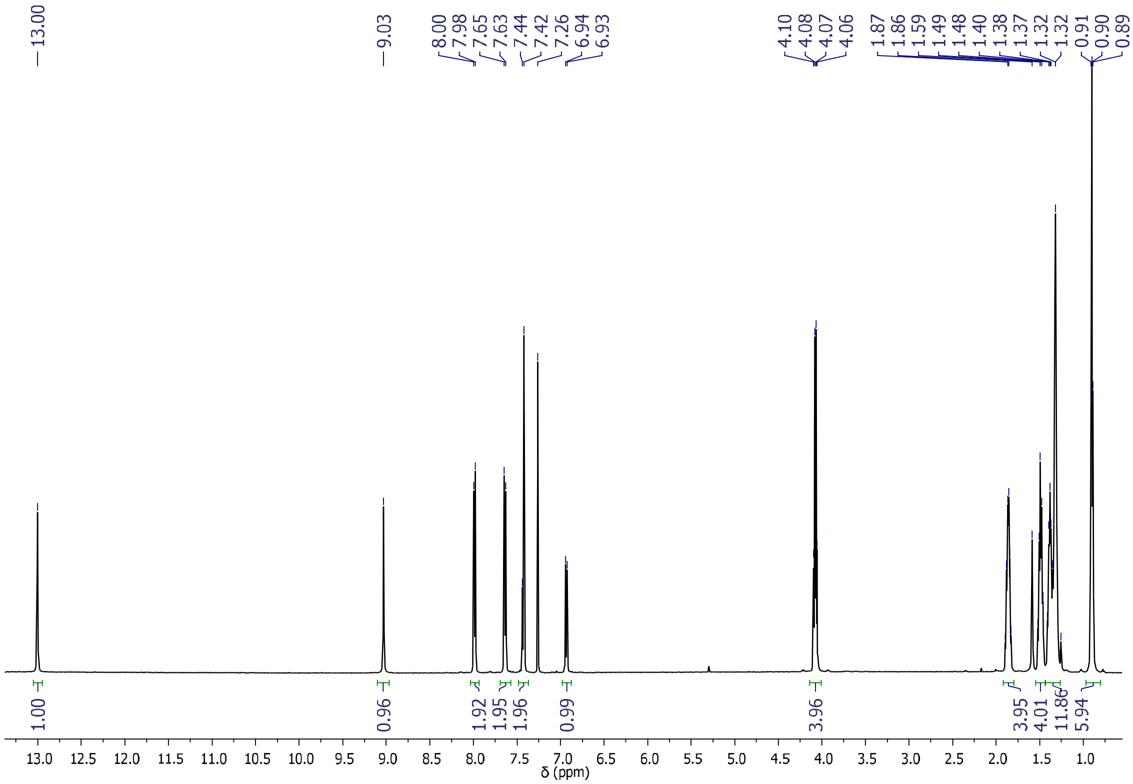

Figure S4. ^{1}H-NMR spectrum of ligand **1**.

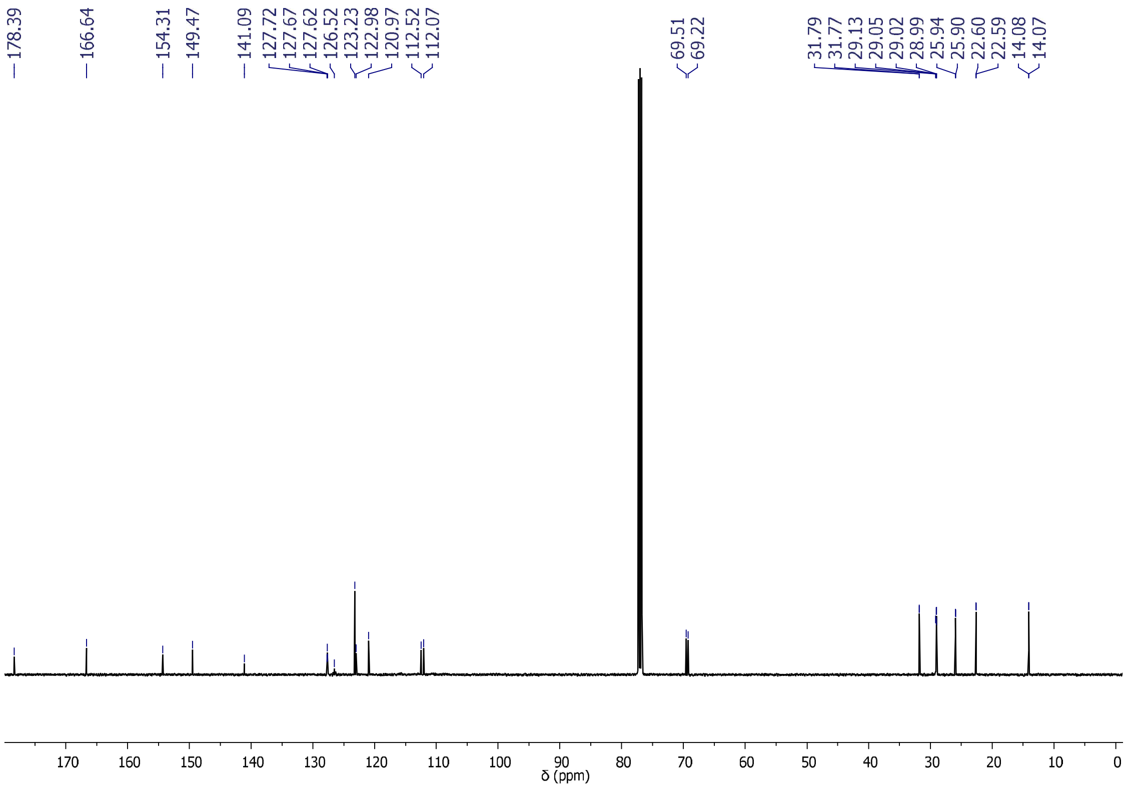

Figure S5. ^{13}C-NMR spectrum of ligand **1**.

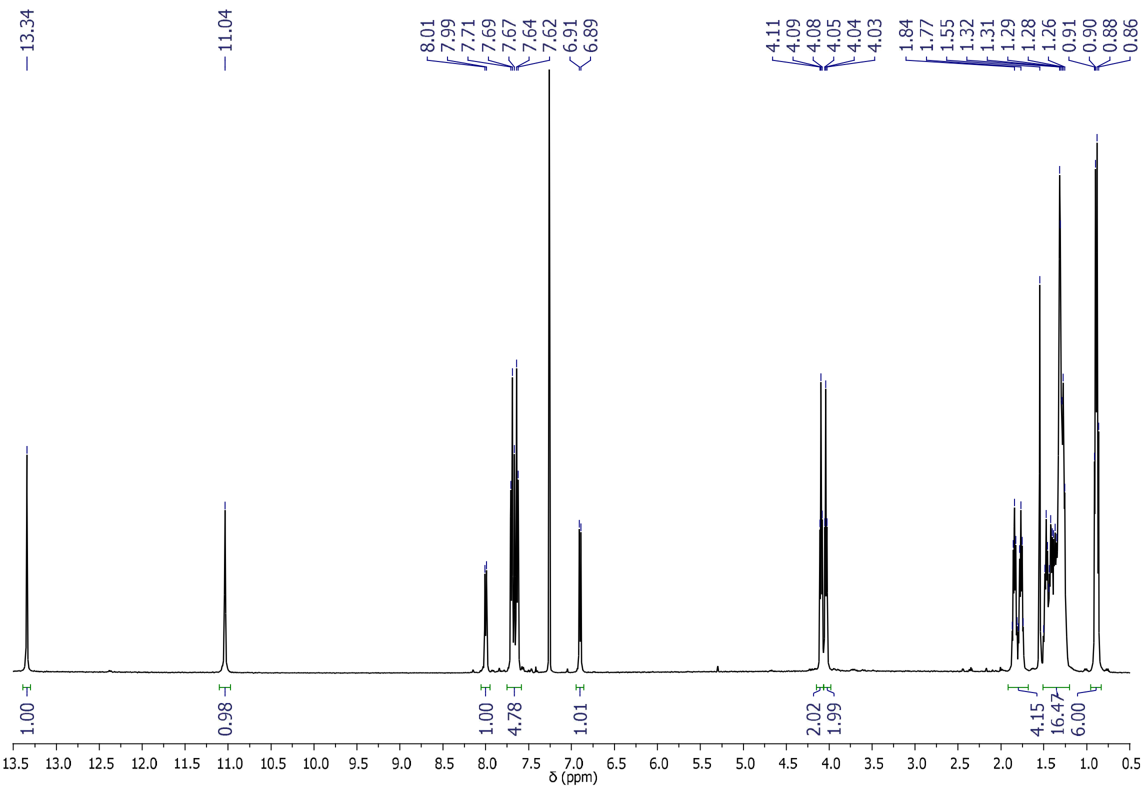

Figure S6. ^{1}H-NMR spectrum of complex **Cu2a**.

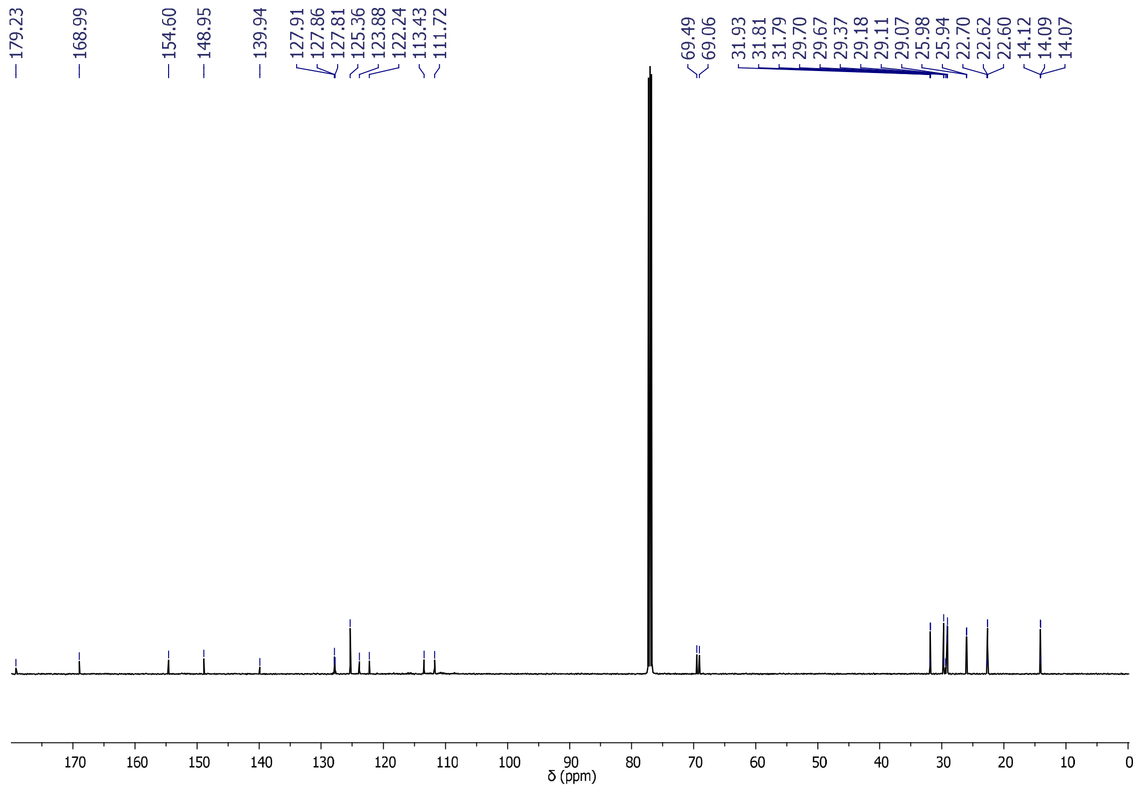

Figure S7. ^{13}C-NMR spectrum of complex **Cu2a**.

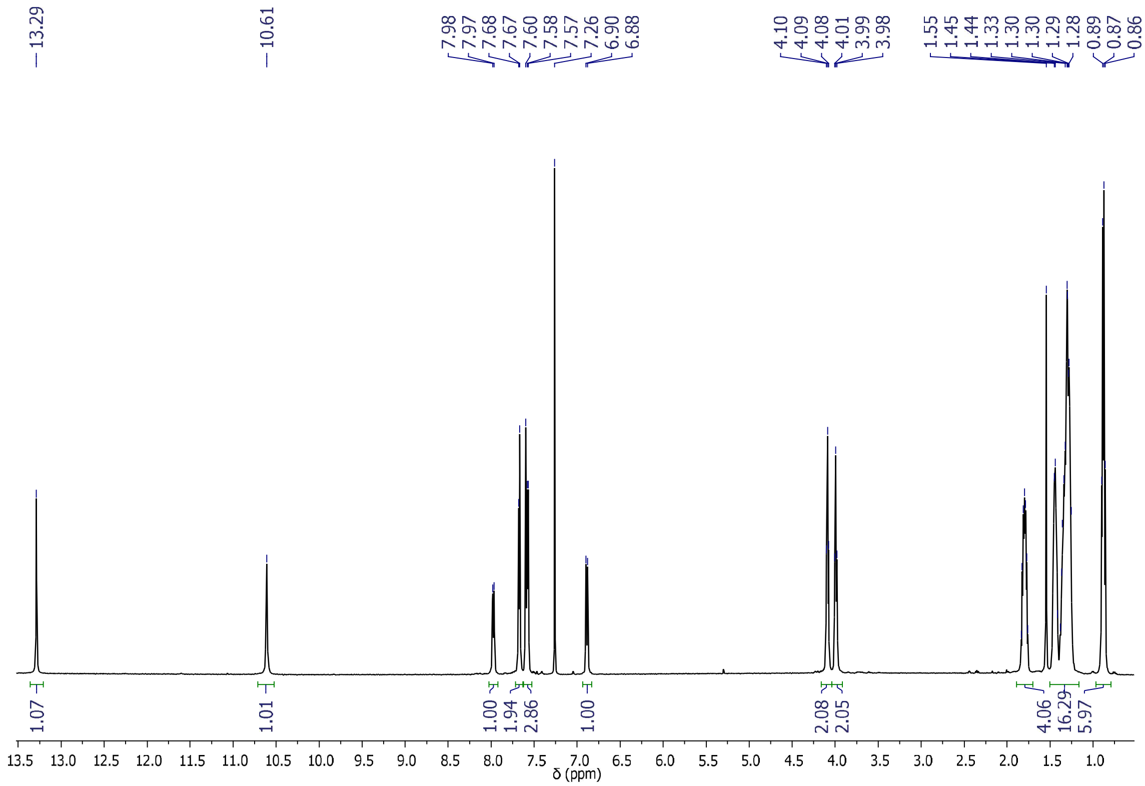

Figure S8. ^{1}H-NMR spectrum of complex **Cu2b**.

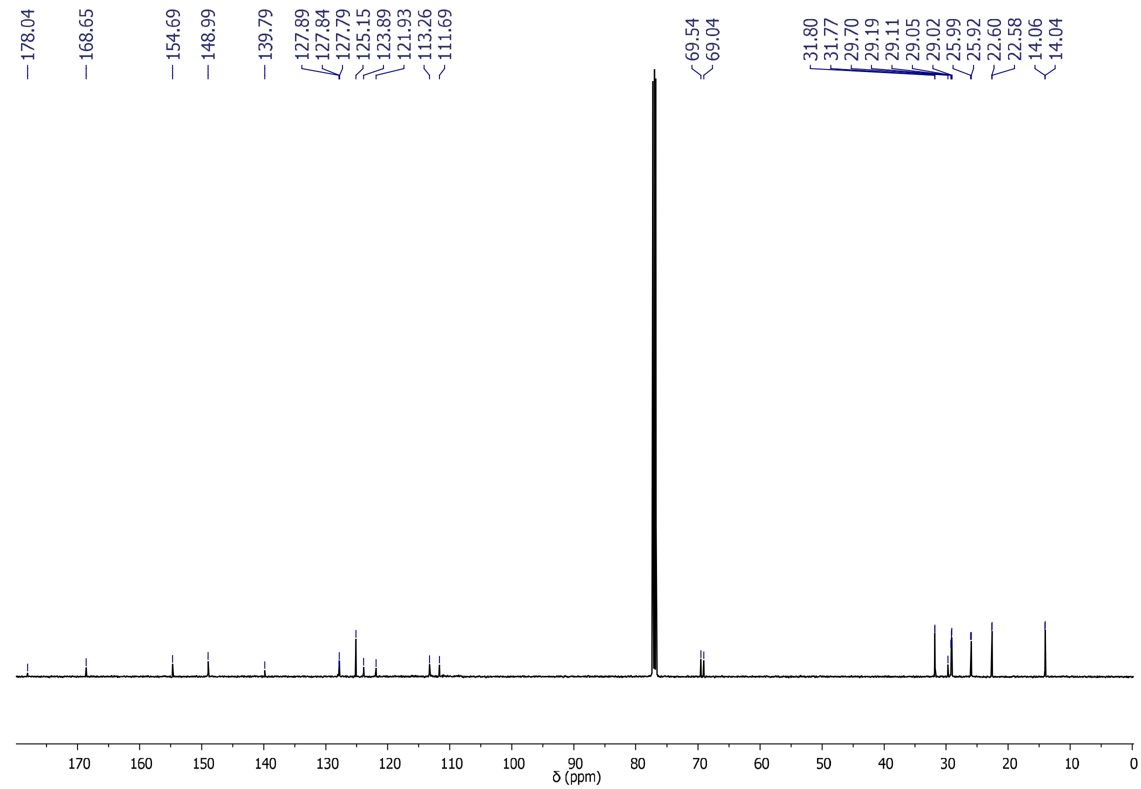

Figure S9. ^{13}C-NMR spectrum of complex **Cu2b**.

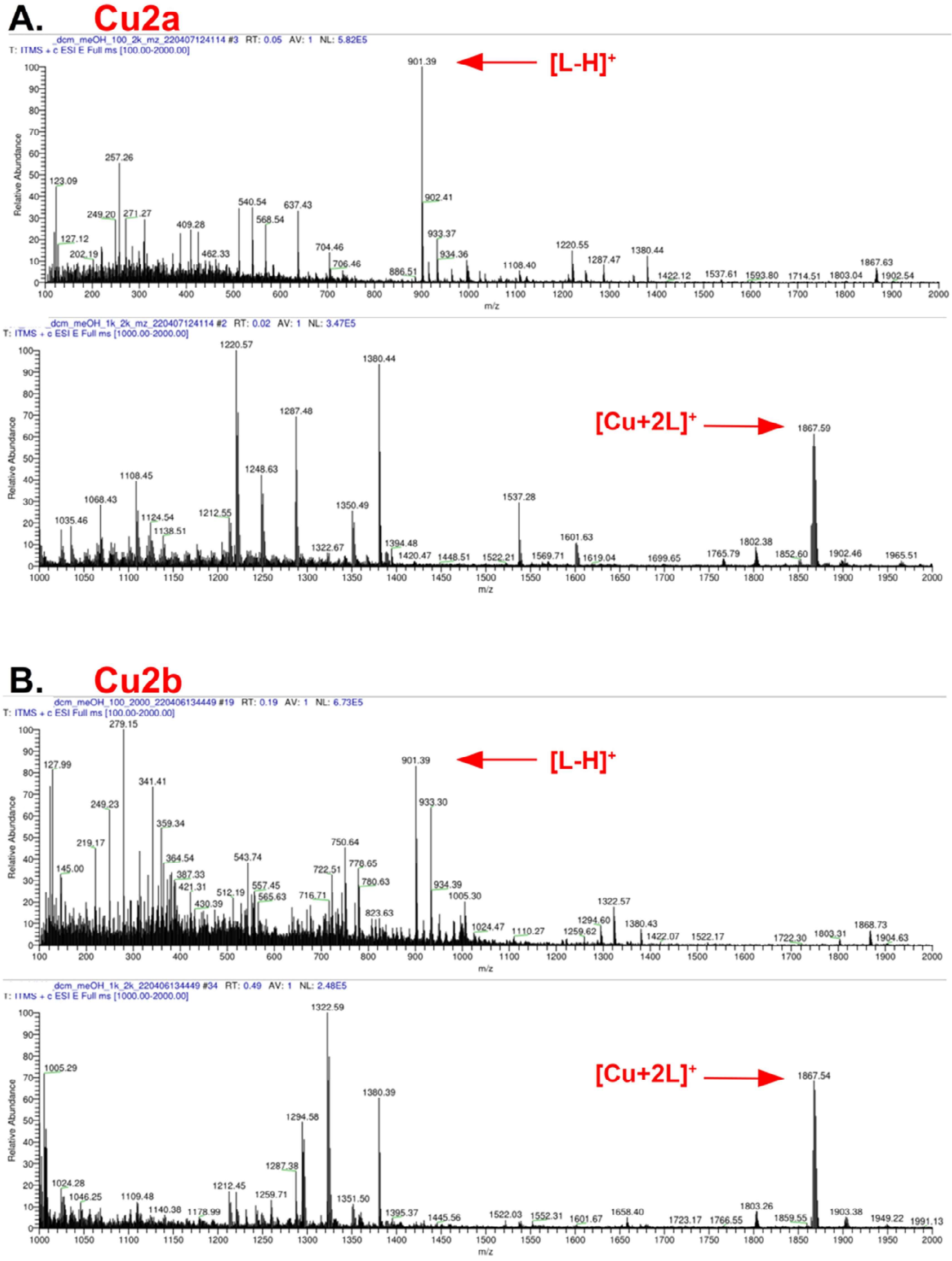

Figure S10. Mass spectrometry identification of $[Cu+2L]^+$ and $[L\text{-}H]^+$ ions of **Cu2a** (A) and **Cu2b** (B) corresponding to calculated m/z.

Generally, the electrical behavior of a material can be investigated by the spectra of two important quantities: the conductivity (the ability of the material to conduct electric current) and the permittivity (as a measure of the electric polarizability of a dielectric material). In this work, the electrical properties of the two copper(I) complexes were analyzed by dielectric spectroscopy and the experimental results are presented by employing the spectra of the complex functions of the dielectric permittivity:

$$\varepsilon^*(\omega) = \varepsilon'(\omega) - i\varepsilon''(\omega) \quad (1)$$

and of the electrical conductivity:

$$\sigma^*(\omega) = \sigma'(\omega) + i\sigma''(\omega) \quad (2)$$

Between the two electric quantities, there is a simple and direct relationship: $\sigma^*(\omega) = i\omega\varepsilon_0\varepsilon^*(\omega)$. The two representations of the dielectric data are complementary and emphasize different aspects of the same processes.

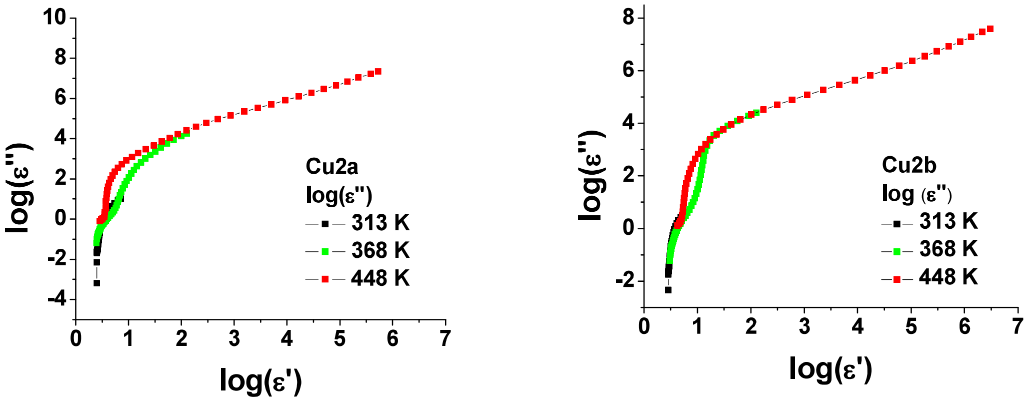

(a) (b)

Figure S11. Overviews: dielectric losses versus dielectric constant for the coppper(I) complexes at different temperatures (T_1=313.15 K, T_2=368.15 K and T_3=448 K): **Cu2a** (a) and **Cu2b** (b).

The activation energy, E_A, for the ionic conductivity mechanism in the mesophase was obtained from the Arrhenius plot corresponding to relation (3) (Figure S12).

$$\sigma_{DC} = \sigma_0 \exp\left(-\frac{E_A}{k_B T}\right), \quad (3)$$

where σ_0 is a pre-exponential factor and k_B is Boltzmann's constant. The activation energy in the LC phase, E_A, calculated from the linear fit of the curve in the corresponding Arrhenius plot is around 1.2 eV for **Cu2a** and 1.5 eV for **Cu2b**, similar to other values reported for metallomesogens showing ionic conduction [1].

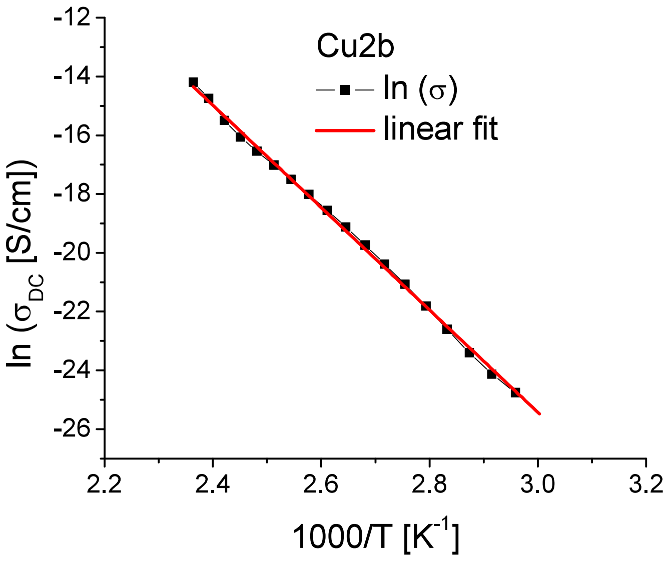

Figure S12. DC conductivity (natural logarithm) versus 1000/T and the linear fitting function for **Cu2b**.

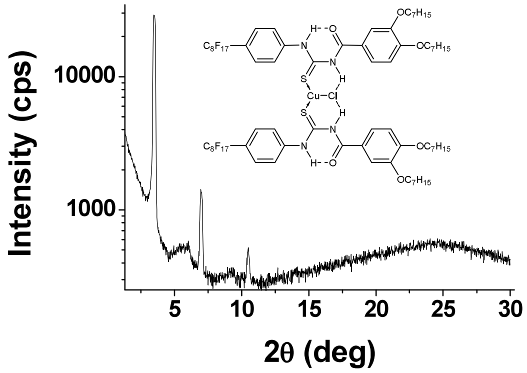

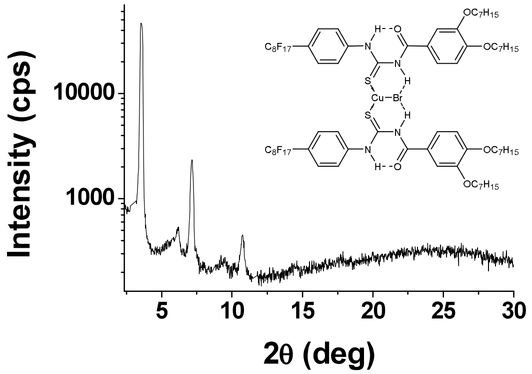

(a) (b)

Figure S13. Powder XRD pattern for complex **Cu2a** (a) and **Cu2b** (b) in the hexagonal columnar mesophase (Col_h) at 388 K recorded on heating.

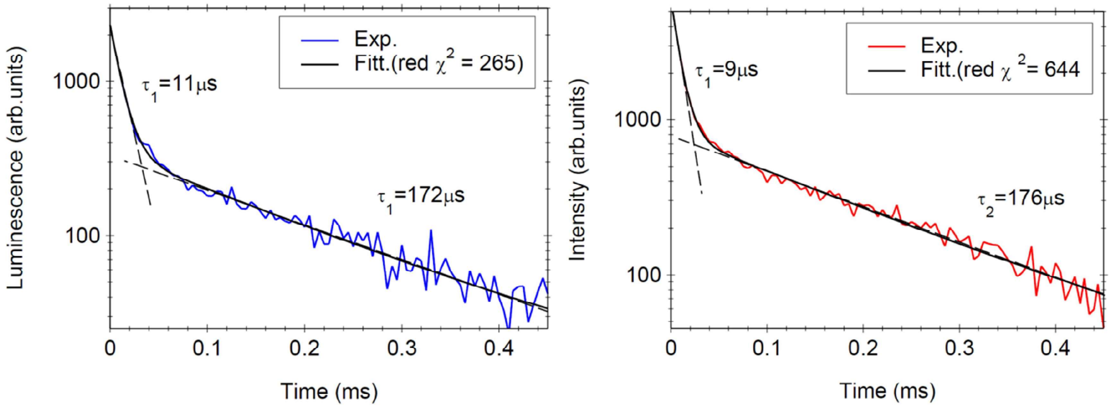

Figure S14. Photoluminescence decay profiles for **Cu1a** (a) and **Cu2b** (b) represented in the log scale, showing the two exponential decays. For the fitting we have used the Origin commercial software and two exponential decay curves; for the goodness of fitting we used the reduced chi-square parameter.

References:

[1]. C. Cuerva, J. A. Campo, M. Cano, R. Schmidt, Nanostructured discotic Pd(II) metallomesogens as one-dimensional proton conductors, *Dalton Trans.*, 2017, 46, 96-105. doi.org/10.1039/C6DT03521C
